# Management of Diarrhea in Young Children in Sub-Saharan Africa: Adherence to World Health Organization Recommendations During the Global Enteric Multisite Study (2007–2011) and the Vaccine Impact of Diarrhea in Africa (VIDA) Study (2015–2018)

**DOI:** 10.1093/cid/ciac926

**Published:** 2023-04-19

**Authors:** Emily L Deichsel, Adama Mamby Keita, Jennifer R Verani, Helen Powell, Leslie P Jamka, M Jahangir Hossain, Joquina Chiquita M Jones, Richard Omore, Alex O Awuor, Samba O Sow, Doh Sanogo, Milagritos D Tapia, Kathleen M Neuzil, Karen L Kotloff

**Affiliations:** Center for Vaccine Development and Global Health, University of Maryland School of Medicine, Baltimore, Maryland, USA; Centre pour le Développement des Vaccins du Mali, Bamako, Mali; Division of Global Health Protection, Centers for Disease Control and Prevention, Nairobi, Kenya; Center for Vaccine Development and Global Health, University of Maryland School of Medicine, Baltimore, Maryland, USA; Center for Vaccine Development and Global Health, University of Maryland School of Medicine, Baltimore, Maryland, USA; Medical Research Council Unit The Gambia at the London School of Hygiene and Tropical Medicine, Banjul, The Gambia; Medical Research Council Unit The Gambia at the London School of Hygiene and Tropical Medicine, Banjul, The Gambia; Kenya Medical Research Institute, Center for Global Health Research, Kisumu, Kenya; Kenya Medical Research Institute, Center for Global Health Research, Kisumu, Kenya; Centre pour le Développement des Vaccins du Mali, Bamako, Mali; Centre pour le Développement des Vaccins du Mali, Bamako, Mali; Center for Vaccine Development and Global Health, University of Maryland School of Medicine, Baltimore, Maryland, USA; Center for Vaccine Development and Global Health, University of Maryland School of Medicine, Baltimore, Maryland, USA; Center for Vaccine Development and Global Health, University of Maryland School of Medicine, Baltimore, Maryland, USA

**Keywords:** diarrhea management, Africa, WHO, IMCI guidelines

## Abstract

**Background:**

Reducing diarrhea-related morbidity and mortality is a global priority, particularly in low-resource settings. We assessed adherence to diarrhea case management indicators in the Global Enteric Multisite Study (GEMS) and Vaccine Impact of Diarrhea in Africa (VIDA) study.

**Methods:**

GEMS (2007–2010) and VIDA (2015–2018) were age-stratified case-control studies of moderate-to-severe diarrhea (MSD) in children aged <5 years. In this case-only analysis, we included children enrolled in The Gambia, Kenya, and Mali. A case with no dehydration received adherent care at home if they were offered more than usual fluids and at least the same as usual to eat. Children with diarrhea and some dehydration are to receive oral rehydration salts (ORS) in the facility. The recommendation for severe dehydration is to receive ORS and intravenous fluids in the facility. Adherent care in the facility included a zinc prescription independent of dehydration severity.

**Results:**

For home-based management of children with MSD and no signs of dehydration, 16.6% in GEMS and 15.6% in VIDA were adherent to guidelines. Adherence to guidelines in the facility was likewise low during GEMS (some dehydration, 18.5%; severe dehydration, 5.5%). The adherence to facility-based rehydration and zinc guidelines improved during VIDA to 37.9% of those with some dehydration and 8.0% of children with severe dehydration.

**Conclusions:**

At research sites in The Gambia, Kenya, and Mali, suboptimal adherence to diarrhea case management guidelines for children aged <5 years was observed. Opportunities exist for improvement in case management for children with diarrhea in low-resource settings.

## Teaser

We assessed adherence to World Health Organization Integrated Management of Childhood Illnesses guidelines for the treatment of diarrhea in sub-Saharan African children enrolled in the Global Enteric Multisite Study and Vaccine Impact of Diarrhea in Africa study.

Diarrhea remains a leading cause of mortality and illness among young children, with more than 500 000 deaths estimated to have occurred globally among children aged <5 years in 2019 [1]. After a diarrheal episode, young children are at an increased risk of repeated episodes, malnutrition, and cognitive delays, which can have long-term consequences [2, 3]. In 2019, diarrhea accounted for more than 45 million disability-adjusted life-years lost in children aged <5 years [1].

In the 1990s, the World Health Organization (WHO) and the United Nations International Children's Emergency Fund developed the Integrated Management of Childhood Illness (IMCI) strategy to reduce child morbidity and mortality and improve growth and development in children aged <5 years [4]. The focus of the guidelines for treating diarrheal disease has been the prevention and treatment of dehydration and promotion of optimal nutrition during a diarrheal episode. In the health center, children are categorized by clinical findings as having none, some, or severe dehydration, and treatment is framed accordingly. Children with some dehydration receive low osmolarity oral rehydration salts (ORS) in the health facility, while those who cannot tolerate oral fluids or have severe dehydration receive initial intravenous (IV) fluids. The home management guidlines for children who have diarrhea with no dehydration and those who have already been rehydrated at the health center recommend extra fluids (including increased breastfeeding) and ORS to prevent dehydration. All children with diarrhea should continue feeding and receive a prescription for zinc. As a component of the IMCI, the WHO Global Strategy on Human Resources for Health: Workforce 2030 [5] encourages countries to use community-based health workers to assess diarrheal disease severity using established criteria; for mildly ill children deemed appropriate for home care, their caretakers are educated on providing appropriate home care for diarrhea as described above [6]. Public health messages in the media and routine health visits are other means for communicating this information to families.

While IMCI recommendations have been adopted by more than 100 countries, adherence has historically been low, particularly in countries with the highest childhood mortality rates [4, 7]. Based on aggregated data from 1985 to 2012, between 35% and 50% of children with diarrhea in low- and middle-income countries received care in accordance with case management guidelines [7, 8]. Greater adherence to diarrhea management guidelines has the potential to improve health outcomes and avert diarrheal deaths [9, 10]. Quantifying adherence to facility and home diarrhea management guidelines for children aged <5 years with diarrhea on a local level will inform the need for further improvements that could reduce childhood mortality.

We analyzed data from the Vaccine Impact on Diarrhea in Africa (VIDA) study (2015–2018) to quantify adherence to recommendations for rehydration during healthcare center visits for moderate-to-severe diarrhea (MSD) and while at home before seeking care. To determine if home practices and current levels of adherence to guidelines had improved over the last decade, we compared these results with adherence during the Global Enteric Multicenter Study (GEMS; 2007–2011), a study that used similar methodology and definitions [11].

## METHODS

### Study Design, Population, and Enrollment

GEMS, a prospective, age-stratified, matched, case-control study, characterized the incidence, etiology, and clinical consequences of MSD among children aged 0–59 months at 7 sites in Africa and Asia [11, 12]. GEMS was conducted over a 36-month period at each site between 2008 and 2012. VIDA, which used study methods similar to those for GEMS to assess MSD after rotavirus vaccine introduction [11, 12], was performed at 3 of the GEMS sites in sub-Saharan Africa—Basse, The Gambia; Siaya County, Kenya; and Bamako, Mali. In addition, Bansang, a demographic surveillance system (DSS) area adjacent to Basse, was added to VIDA to ensure sufficient sample sizes. Powell et al [13] provide an in-depth discussion of relevant differences between GEMS and VIDA methodologies.

At each site, children aged 0–59 months with MSD were enrolled from sentinel health centers (SHCs) where the DSS population received care. Eligible children were those with a new episode of diarrhea (≥3 loose stools within 24 hours, onset within the past 7 days after ≥7 diarrhea-free days) and at least 1 of the following: sunken eyes, decreased skin turgor, IV hydration administered or prescribed, dysentery, or admission/recommendation for admission to the hospital [11, 14].

### Clinical and Epidemiological Data Collection

We collected demographic, epidemiological, and clinical information at enrollment and performed anthropometric measurements using standardized methods [11]. Interviews of caretakers inquired about the child's signs and symptoms and how the illness was managed at home prior to seeking care at the SHC. Clinical findings, diagnosis, and medical management, including fluids or medications that were prescribed or administered, were documented upon presentation to the SHC and throughout the SHC stay in the outpatient or inpatient facility.

### Population and Outcome Definitions

Our study population for this analysis included MSD cases enrolled in the GEMS and VIDA study from the aforementioned sites. Cases were included in this analysis if complete data documenting the following were recorded: caretaker report of home management of the diarrheal illness, including use of rehydration fluids and continued feeding, prior to visiting the SHC; clinical findings elicited by medical history or physical examination upon enrollment at the SHC that allow determination of dehydration status according to IMCI criteria; and treatment prescribed or received for diarrhea at the SHC (zinc, ORS, and/or IV fluid). Children missing data on any of these variables were excluded.

Dehydration definitions and recommended treatment based on WHO IMCI guidelines are summarized in Table 1 [15, 16]. We considered a child adherent if they received all of the recommended treatment(s) for their level of dehydration. For example, a child with severe dehydration was considered to have received care adherent to facility-based treatment guidelines if that child received both IV fluids and ORS at the facility and either prescription or administration of zinc. Neither frequency of IV fluids nor ORS were considered; rather a single administration was sufficient to meet the criteria for adherence to the respective treatment.

**Table 1.** Classification of Dehydration and Treatment Recommendations for Children With Diarrhea Adapted for Analysis

Dehydration Classification	Signs	Home Treatment Guidelines	Clinic Treatment Guidelines
Severe	2 of the following:	• NA (seek care at clinic)	• Intravenous fluids administration,
	• Lethargic or unconscious		• ORS administration, and
	• Sunken eyes		• Zinc supplementation (prescription)
	• Unable to drink or drinks poorly		
	• Skin pinch goes back very slowly		
Some	2 of the following:	• NA (seek care at clinic)	• ORS administration, and
	• Restless, irritable		• Zinc supplementation (prescription)
	• Sunken eyes		
	• Drinks eagerly, thirsty		
	• Skin pinch goes back slowly		
None	Not enough signs to classify as some or severe dehydration	• Oral hydration (more than usual), and	• Zinc supplementation (prescription)
		• Continued feeding (at least as much as usual)	

Table adapted from World Health Organization Integrated Management of Childhood Illness handbook [16].

Based on guidelines, a child with diarrhea and signs of some or severe dehydration should receive rehydration treatment at the health center. Those with diarrhea and no signs of dehydration only require rehydration, which can be administered at the home.

Abbreviations: NA, not applicable; ORS, oral rehydration salts.

We described the adherence to home and facility-based management guidelines by dehydration severity, study, and site. Home management occurred prior to enrollment and was defined by 2 binary variables: continued feeding (offering the same or more than usual) and more than usual fluids offered. Information on treatment at home was collected at enrollment through caregiver report. Facility-based management was defined by zinc prescription (binary) and fluids given (categorical; none, ORS only, IV only, ORS and IV). Information on treatment at the health facility was collected at discharge from inpatient or outpatient sites by SHC study staff. We described each of these parameters for GEMS and VIDA by site. Adherence to home- and facility-based management guidelines are heavily influenced by resources that could vary considerably by time period, country, and even facility. We, therefore, refrained from making population-level inferences based on the data presented here and focused on the findings for the study population only. All analyses were conducted using R version 4.2.0.

This study was approved by the ethical review committees at the University of Maryland, Baltimore (HP-00062472), the Centers for Disease Control and Prevention (CDC) (reliance agreement 6729), The Gambia Government/Medical Research Council/Gambia at the London School of Hygiene & Tropical Medicine (1409), the Comité d'Ethique de la Faculté de Médecine, de Pharmacie, et d'Odonto-Stomatologie, Bamako, Mali (no number), and the Kenya Medical Research Institute Scientific & Ethics Review Unit in Siaya County, Kenya (SSE 2996). Informed, written consent was obtained from all participants prior to initiation of study procedures.

## RESULTS

### Study Population

Among the 4538 MSD cases enrolled in GEMS from the sites in The Gambia, Kenya, and Mali, 3 cases were excluded from this analysis due to missing data, leaving 4535 included. Of included children, 26.0% (1177) had a caretaker with more than a primary school education and 4.1% (184) and 83.1% (3768) lived in homes with access to improved sanitation and improved water sources, respectively (Table 2). Five percent (235) of children with MSD had severe acute malnutrition (SAM; mid-upper arm circumferance < 11.5 cm).

**Table 2.** Enrolled Cases, Children Aged 0–59 Months With Moderate-to-Severe Diarrhea by Site and Study: Global Enteric Multisite Study, 2007–2011 and Vaccine Impact of Diarrhea in Africa Study, 2015–2018

	Total		The Gambia		Kenya		Mali	
Characteristics	GEMS	VIDA	GEMS	VIDA	GEMS	VIDA	GEMS	VIDA
Total	4535	4791	1027	1641	1475	1547	2033	1603
Age, m								
ȃ0–11	1798 (39.6%)	1711 (35.7%)	399 (38.9%)	531 (32.4%)	672 (45.6%)	586 (37.9%)	727 (35.8%)	594 (37.1%)
ȃ12–23	1546 (34.1%)	1677 (35.0%)	454 (44.2%)	601 (36.6%)	410 (27.8%)	525 (33.9%)	682 (33.5%)	551 (34.4%)
ȃ24–59	1191 (26.3%)	1403 (29.3%)	174 (16.9%)	509 (31.0%)	393 (26.6%)	436 (28.2%)	624 (30.7%)	458 (28.6%)
Female	2004 (44.2%)	2231 (46.6%)	458 (44.6%)	762 (46.4%)	636 (43.1%)	705 (45.6%)	910 (44.8%)	764 (47.7%)
Caregiver education, more than primary	1177 (26.0%)	1617 (33.8%)	68 (6.6%)	245 (14.9%)	803 (54.4%)	1024 (66.2%)	306 (15.1%)	348 (21.7%)
Improved sanitation^a^	184 (4.1%)	3008 (62.8%)	28 (2.7%)	721 (43.9%)	89 (6.0%)	700 (45.2%)	67 (3.3%)	1587 (99.0%)
Improved water source^a^	3768 (83.1%)	4033 (84.2%)	860 (83.7%)	1329 (81.0%)	875 (59.3%)	1102 (71.2%)	2033 (100%)	1602 (99.9%)
Dehydration status^b^								
ȃSevere	796 (17.6%)	679 (14.2%)	144 (14.0%)	148 (9.0%)	407 (27.6%)	414 (26.8%)	245 (12.1%)	117 (7.3%)
ȃSome	3311 (73.0%)	3658 (76.4%)	671 (65.4%)	1154 (70.3%)	1024 (69.4%)	1050 (67.9%)	1616 (79.5%)	1454 (90.7%)
ȃNone	428 (9.4%)	454 (9.5%)	212 (20.6%)	339 (20.7%)	44 (3.0%)	83 (5.4%)	172 (8.5%)	32 (2.0%)
Dysentery	635 (14.0%)	734 (15.3%)	221 (21.5%)	470 (28.6%)	169 (11.5%)	204 (13.2%)	245 (12.1%)	60 (3.7%)
Acute malnutrition^c^								
ȃSevere	235 (5.2%)	145 (3.0%)	69 (6.7%)	78 (4.8%)	80 (5.4%)	33 (2.1%)	86 (4.2%)	34 (2.1%)
ȃModerate	472 (10.4%)	468 (9.8%)	141 (13.7%)	218 (13.3%)	124 (8.4%)	89 (5.8%)	207 (10.2%)	161 (10.0%)

Abbreviations: GEMS, Global Enteric Multisite Study; VIDA, Vaccine Impact of Diarrhea in Africa study.

Definition based on Join Monitoring Program definitions.

Definition based on World Health Organization Integrated Management of Childhood Illness guidelines (see Table 1).

Defined using mid-upper arm circumference. Severe: <11.5 cm, moderate:≥ 11.5 and <12.5 cm.

Of the 4840 children with MSD enrolled at the 3 VIDA sites, 4791 were included in this analysis, and 49 were excluded for missing data. Among these MSD cases, 33.8% (1617) of primary caretakers had more than a primary school education and 62.8% (3008) and 84.2% (4033) reported having improved sanitation and an improved water source, respectively. Three percent (145) of enrolled cases in VIDA had SAM. Additional descriptions of participants are shown by study site in Table 2.

In GEMS, 17.6% (796) of enrolled cases had severe dehydration, 73.0% (3311) had some dehydration, and 9.4% (428) had no signs of dehydration. The numbers were similar in VIDA, 14.2% (679) of enrolled children had severe dehydration, 76.4% (3658) had some dehydration, and 9.5% (454) had no signs of dehydration (Table 2).

### Adherence to WHO Guidelines

We evaluated the adherence to rehydration guidelines for case management of diarrhea at home prior to the SHC visit by caretaker interview at the time of the child's study enrollment at the SHC (Table 3). Among the 428 children enrolled in GEMS with MSD and no signs of dehydration at presentation, 16.6% (71) had been offered both additional fluids and continued feeding at home in accordance with IMCI guidelines. Less than half (40.9%; 175) were offered more than usual to drink, and 53.3% (228) were offered as much as usual to eat, as recommended. In VIDA, among the 454 children with MSD and no signs of dehydration, 15.6% (71) received home case management for diarrhea according to IMCI guidelines. Only 28.6% (130) were offered more than usual to drink, while 65.4% (297) received continued feeding (at least as much as usual). A little more than half of those who sought care with no signs of dehydration received the recommended zinc prescription.

**Table 3.** Adherence to World Health Organization Rehydration Guidelines by Severity of Dehydration Among Global Enteric Multisite Study (2007–2011) and Vaccine Impact of Diarrhea in Africa Study (2015–2018) Cases

		No Dehydration		Some Dehydration		Severe Dehydration	
		GEMS N = 428 n (%)	VIDA N = 454 n (%)	GEMS N = 3309 n (%)	VIDA N = 3658 n (%)	GEMS N = 795 n (%)	VIDA N = 679 n (%)
Adherent to WHO rehydration guidelines at home	Yes	71 (16.6%)	71 (15.6%)	NA	NA	NA	NA
ȃSince the child developed diarrhea, how much have you been offering the child to drink?	More than usual	175 (40.9%)	130 (28.6%)	2172 (65.6%)	2418 (66.1%)	300 (37.7%)	273 (40.2%)
ȃSince the child developed diarrhea, how much have you been offering the child to eat?	At least as much as usual	228 (53.3%)	297 (65.4%)	1057 (31.9%)	1476 (40.3%)	199 (25.0%)	235 (34.6%)
Adherent to WHO rehydration guidelines in a facility	Yes	29 (6.8%)	245 (54.0%)	614 (18.5%)	1388 (37.9%)	44 (5.5%)	54 (8.0%)
ȃZinc prescribed	Yes	29 (6.8%)	245 (54.0%)	622 (18.8%)	1668 (45.6%)	187 (23.5%)	433 (63.8%)
ȃORS administered	Yes	51 (11.9%)	126 (27.8%)	1104 (33.3%)	1777 (48.6%)	414 (52.0%)	453 (66.7%)
ȃIntravenous rehydration administered	Yes	16 (3.7%)	3 (0.7%)	408 (12.3%)	220 (6.0%)	206 (25.9%)	115 (16.9%)
ȃORS prescribed	Yes	360 (84.1%)	402 (88.5%)	3124 (94.4%)	3627 (99.2%)	721 (90.6%)	655 (96.5%)

Shaded cells indicate a treatment is required by WHO rehydration guidelines for the given degree of dehydration.

Abbreviations: GEMS, Global Enteric Multisite Study; NA, not applicable; ORS, oral rehydration salts; VIDA, Vaccine Impact of Diarrhea in Africa study; WHO, World Health Organization.

Among children enrolled in GEMS who had some dehydration, 18.5% (614) received both ORS and a zinc prescription at the facility (Table 3). An even smaller proportion, 5.5% (44), of children with severe dehydration received recommended care, which included IV and ORS administration, along with a zinc prescription. While 52.0% (414) of severely dehydrated children received ORS in the health facility, only 25.9% (206) received an IV as indicated based on rehydration status. Among VIDA participants with some dehydration, 37.9% (1388) received both a zinc prescription and ORS administered at the facility in accordance with the WHO guidelines. Of VIDA participants with severe dehydration, 63.8% (433) received the recommended zinc prescription and 66.7% (453) received the recommended ORS administration in a health facility, while 16.9% (115) received IV rehydration. Eight percent (54) of participants with severe dehydration received the recommended zinc treatment and administration of both ORS and an IV. A prescription for ORS was given to almost all participants who presented with MSD during GEMS and VIDA, with the highest prescription rates among those with some and severe dehydration during VIDA (99.2% and 96.5%, respectively).

### Changes in Adherence to WHO Rehydration Guidelines From GEMS to VIDA, Overall and by Site

**Figure 1.**
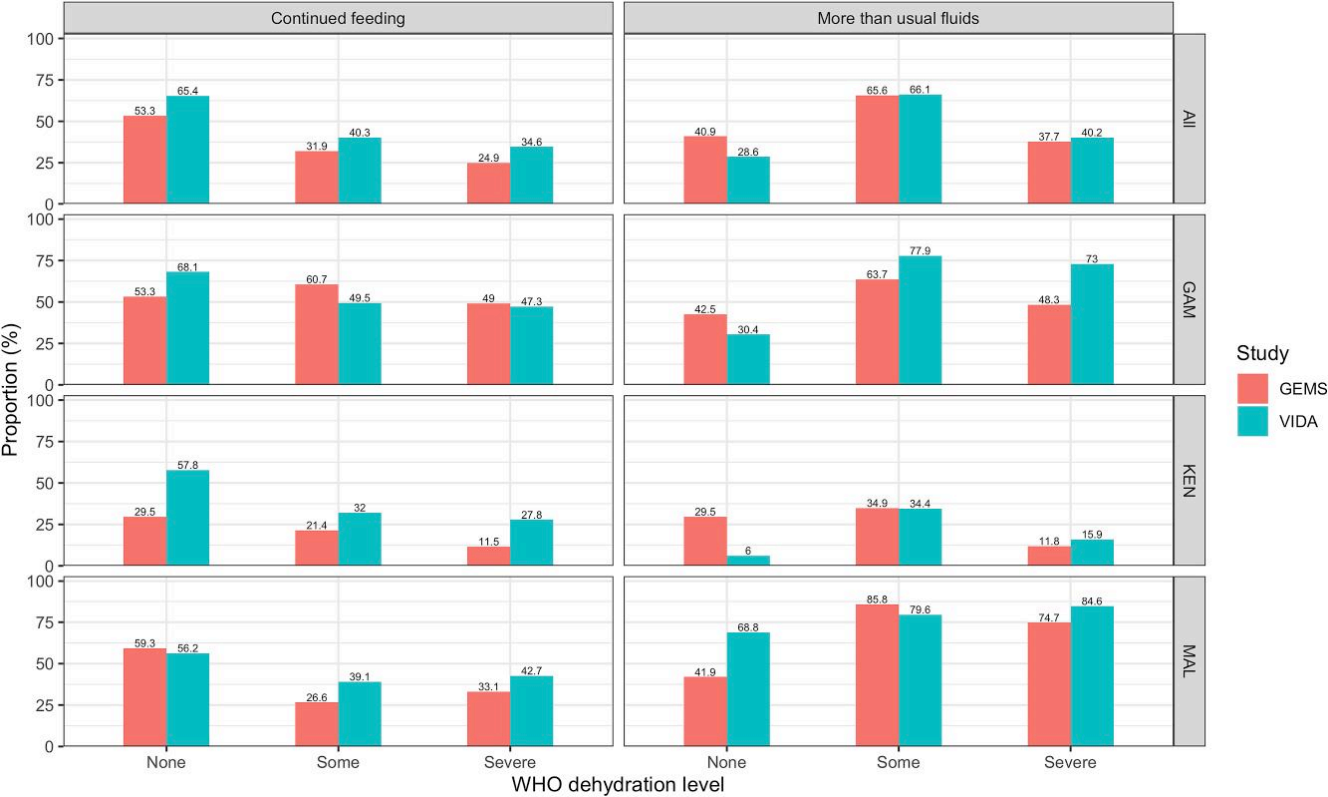
Adherence to WHO IMCI home management guidelines by WHO dehydration level, site, and study (GEMS, 2007–2011 and VIDA, 2015–2018). Abbreviations: GEMS, Global Enteric Multisite Study; VIDA, Vaccine Impact of Diarrhea in Africa study; WHO, World Health Organization.

**Figure 2.**
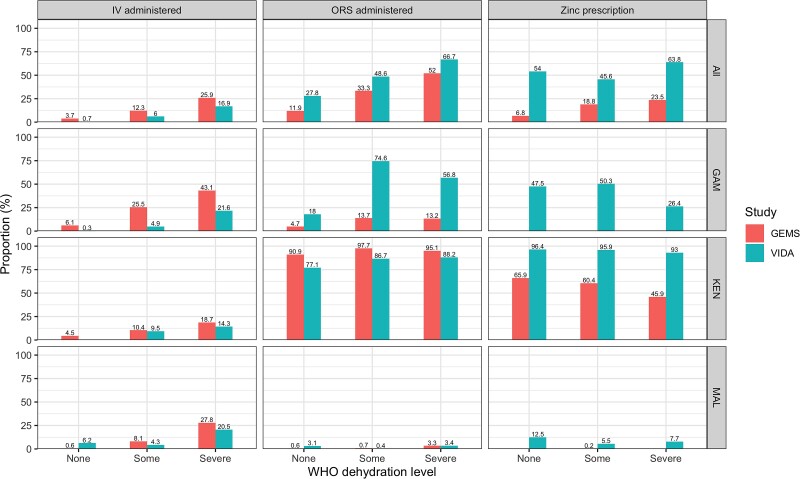
Adherence to WHO IMCI facility-based management guidelines by WHO dehydration level, site, and study (GEMS, 2007–2011 and VIDA, 2015–2018). Abbreviations: GAM, The Gambia; GEMS, Global Enteric Multisite Study; IV, intravenous; KEN, Kenya; MAL, Mali; ORS, oral rehydration salts; VIDA, Vaccine Impact of Diarrhea in Africa study; WHO, World Health Organization.

We evaluated adherence overall, by site, by study, and by specific guideline components (Figures 1 and 2; Supplementary Tables 1, 2, and 3). Overall, adherence to guidelines in the facility improved during the VIDA study compared with GEMS, although the improvement was minimal for severe dehydration, with a 2.5% increase in adherence across sites (Table 4). While the facility adherence was generally better, the home adherence to guidelines did not improve during VIDA, with 1 exception: a 12.0% increase in home management adherence from GEMS to VIDA at the Mali site.

**Table 4.** Adherence to World Health Organization Integrated Management of Childhood Illness Rehydration Guidelines During Vaccine Impact of Diarrhea in Africa Study (2015–2018) at Home and in a Facility by Site and Dehydration Status and Change in Adherence Compared With the Global Enteric Multisite Study (2007–2011)

	Adherence to Integrated Management of Childhood Illness Guidelines During Vaccine Impact of Diarrhea in Africa Study			Change in Percent Adherence from the Global Enteric Multisite Study^a^		
Guideline	No Dehydration	Some Dehydration	Severe Dehydration	No Dehydration	Some Dehydration	Severe Dehydration
The Gambia						
	N = 339 n (%)	N = 1154 n (%)	N = 148 n (%)	NA	NA	NA
Home	59 (17.4%)	NA	NA	0.4%	NA	NA
Facility	161 (47.5%)	517 (44.8%)	10 (6.8%)	47.5%	44.8%	6.8%
Kenya						
	N = 83	N = 1050	N = 414	NA	NA	NA
Home	2 (2.4%)	NA	NA	−2%	NA	NA
Facility	80 (96.4%)	871 (83.0%)	44 (10.6%)	30.5%	23.0%	−0.2%
Mali						
	N = 32	N = 1454	N = 117	NA	NA	NA
Home	10 (31.2%)	NA	NA	12.0%	NA	NA
Facility	4 (12.5%)	0 (0.0%)	0 (0.0%)	12.5%	0%	0%
All sites						
	N = 454	N = 3658	N = 679	NA	NA	NA
Home	71 (15.6%)	NA	NA	−1.0%	NA	NA
Facility	245 (54.0%)	1388 (37.9%)	54 (8.0%)	47.2%	19.4%	2.5%

Negative number indicates decrease in adherence; positive number indicates an increase.

Abbreviation: NA, not applicable.

Adherence to specific guideline components varied more markedly than the overall adherence (Figures 1 and 2, Table 3). Before seeking care at a SHC, fewer children who presented with no dehydration in VIDA (28.6%; 130) compared with GEMS (40.9%; 175) were offered more than usual to drink. Conversely, there was an increase in VIDA compared with GEMS across all dehydration categories in the proportion of children (all sites combined) who were offered at least as much as usual to eat. The site differences were more variable. For children with no dehydration, the Mali site saw an increase from 41.9% (72) to 68.8% (22) in the proportion of children who were offered more fluids than usual (Supplementary Table 1) and a small decrease from 59.3% (102) to 56.2% (18) in the proportion who received continued feeding.

When considering the facility-based guidelines, a higher proportion of severely dehydrated participants from the VIDA study received ORS (66.7%) and/or zinc prescriptions (63.8%), while a smaller proportion received IV fluids (16.9%) compared with GEMS (52.0% and 23.5% for ORS and/or zinc and 25.9% for IV fluids, respectively; Table 3). The largest improvement in adherence to facility-based guidelines was in children with no dehydration, where 6.8% (29) in GEMS and 54.0% (245) in VIDA received a zinc prescription as recommended. The same pattern was observed in Mali (Supplementary Table 1) and The Gambia (Supplementary Table 2) for the individual components of the facility-based guidelines. In Kenya, the proportion who received recommended ORS decreased between GEMS and VIDA, while the proportion at other sites increased (Supplementary Table 3).

## DISCUSSION

In this study, we assessed the adherence to WHO guidelines for the management of diarrhea in sub-Saharan Africa. Using data from 2 large diarrhea case-control studies conducted from 2007 to 2018, this analysis shows suboptimal adherence to IMCI diarrhea case management guidelines at research sites in The Gambia, Kenya, and Mali. We did not find evidence for improved home management from GEMS (2007–2010) to the VIDA study (2015–2018). The data demonstrate some improvement in facility-based rehydration and zinc prescription adherence across these 2 studies. Additionally, adherence differed by diarrhea severity. Unfortunately, the sites in our study demonstrated lower adherence for diarrhea with severe dehydration compared with some or no dehydration.

Our findings of low adherence to guidelines are similar to those from other recent studies that showed minimal improvements since the development and implementation of IMCI guidelines in the late 1990s and early 2000s [7, 17]. For example, Black et al estimated that 44% of children with diarrhea globally received ORS and 12% received zinc for treatment in 2015 [9]. In 2017, the Local Burden of Disease Diarrhea Collaborators similarly estimated that 49% of children did not receive ORS or home rehydration solution during a diarrheal episode [8].

IMCI guidelines added zinc for the treatment of diarrhea in 2004. While the Kenya site had started to implement zinc during GEMS, no caregiver or facility reported any zinc usage in either The Gambia or Mali during GEMS, illustrating the substantial lag that can occur between a change in international guidelines and implementation into clinical practice. While the opportunity for further progress remains, these results document substantial improvements in the implementation of zinc prescriptions by the start of the VIDA study in 2015. However, the effect of national policies to increase coverage with recommended treatments is inconsistent across settings, as illustrated by the dramatic differences in zinc usage among the 3 study sites [18]. Efforts to promote ORS and zinc for diarrhea treatment in some settings demonstrate progress and that high coverage is possible but requires a multipronged approach that includes supply management, engaging the caregiver and community, and educating health facility staff [19].

Most diarrhea is not severe and can be managed safely by a caregiver at home with ORS or excess fluids, along with continued feeding for children with diarrhea and no signs of dehydration or dysentery [6, 16, 20]. Data tracking adherence to home diarrhea case management guidelines, however, are limited. A multicountry (including Kenya and Mali, but not The Gambia) demographic and health survey analysis in 2004 was consistent with our study's findings of low home-based adherence to recommendations for treatment at home [21]. Few countries in sub-Saharan Africa exceed 50% coverage of increased fluids or continued feeding for children with diarrhea who are managed at home. The same analysis reports that rates of increased fluid administration at home are higher among children who eventually seek care at a health facility, such as in the present analysis. However, continued feeding is the same or slightly less among those who eventually seek care [21].

Adherence to IMCI diarrhea home- and facility-based case management guidelines is low despite decades of varied efforts directed at diarrhea control programs [22]. Improving home management is particularly challenging, as educational programs must be tailored to the targeted community and designed to reach parents, caregivers, and other decision-makers, including traditional healers [23]. Qualitative studies point to the social context in which home management is dependent on the caregiver's perceived cause of diarrhea and the fact that additional fluids did not stop the diarrhea [23, 24]. Other studies point to inadequate education [25], lack of understanding of childhood illness, and competing priorities [26]. In our population of children who did seek care for their illness at a health facility, caregivers may not have administered care at home despite recommendations [7]. In a health facility setting, barriers include limited availability and accessibility of ORS and IV rehydration and high cost of supplies, as well as the training and influence of health center staff [27]. Several studies indicate home- and facility-based management implementation was successful where caregivers were able to access knowledge about diarrhea treatment and oral rehydration therapy and where healthcare professionals could be an important influence on this implementation [27, 28].

Our analysis has some limitations. First, the case definition for GEMS and VIDA only includes medically attended moderate-to-severe diarrhea; consequently, the results are not representative of case management of individuals with less severe diarrhea, who are less likely to receive ORS while at a health facility [29]. However, moderate-to-severe diarrhea is linked to a higher risk of diarrhea-associated mortality and other sequalae, and thus appropriate management of MSD is a priority. Second, home management was only assessed in children seeking care for MSD, possibly representing a select population who were not given guideline-adherent management in the home. Third, these results are from a research setting with additional funding support and oversight, and so the generalizability to other sites is uncertain. However, we believe the presence of the GEMS and VIDA study at these sites would have a positive impact on case management and not the reverse, and thus it is possible that our study overestimated adherence to treatment guidelines, as suggested by lower estimates of ORS coverage in Bamako published elsewhere [18].

## CONCLUSIONS

A large gap exists between diarrheal treatment recommendations and their application by healthcare workers and caregivers in the community. Opportunities exist for improvement in case management for children with moderate-to-severe diarrhea in low-resource settings, and these improvements could reduce mortality and malnutrition. Site-specific sensitization interventions may be needed to improve adherence to WHO diarrhea management guidelines. Implementation-based interventions should consider local knowledge, behavior, and infrastructure.

## Supplementary Data

Supplementary materials are available at *Clinical Infectious Diseases* online. Consisting of data provided by the authors to benefit the reader, the posted materials are not copyedited and are the sole responsibility of the authors, so questions or comments should be addressed to the corresponding author.

## Supplementary Material

ciac926_Supplementary_Data

## Notes

***Acknowledgments.*** The authors express their deep gratitude to the families who participated in these studies, the clinical and field staff for their exceptional hard work and dedication, and to the physicians, administration, and health officials at every site who generously provided facilities and support for the conduct of the study. They are grateful to Catherine Johnson, Chris Focht, and Nora Watson at the Emmes Company, LLC for expert data management and reporting. Special thanks go to Carl Kirkwood, Duncan Steele, and Anita Zaidi at the Bill & Melinda Gates Foundation for helpful oversight, and to the following members of their International Scientific Advisory Committee for providing insightful comments and guidance: Janet Wittes (Chair), George Armah, John Clemens, Christopher Duggan, Stephane Helleringer, Ali Mokdad, James Nataro, and Halvor Sommerfelt. This supplement is sponsored by the Center for Vaccine Development and Global Health (CVD) at the University of Maryland School of Medicine, Baltimore (UMB).

***Financial support*.** This work was funded by the Bill & Melinda Gates Foundation (GEMS, OPP38874 VIDA, OPP1111236). E. L. D. received support from a National Institutes of Health T32 training grant through the University of Maryland School of Medicine Center for Vaccine Development and Global Health (T32AI007524).

***Supplement sponsorship.*** This supplement was sponsored by the Bill & Melinda Gates Foundation. This study is based on research funded in part by grants from the Bill & Melinda Gates Foundation (OPP1111236/OPP1116751).
