# Supplementary material for: Management of Diarrhea in Young Children in Sub-Saharan Africa: Adherence to World Health Organization Recommendations During the Global Enteric Multisite Study (2007–2011) and the Vaccine Impact of Diarrhea in Africa (VIDA) Study (2015–2018)
Source: Clin Infect Dis. 2023 Apr 19;76(Suppl 1):S23–31. doi: 10.1093/cid/ciac926 (PMC10116557; doi:10.1093/cid/ciac926)
Supplement: ciac926_Supplementary_Data [file ciac926_supplementary_data.docx]

**Management of diarrhea in young children in sub-Saharan Africa: Adherence to World Health Organization recommendations during the GEMS (2007–2011) and VIDA study (2015–2018)**

Emily L. Deichsel, Adama Mamby Keita, Jennifer R. Verani, Helen Powell, Leslie P. Jamka, M. Jahangir Hossain, Joquina Chiquita M. Jones, Richard Omore, Alex O. Awuor, Samba O. Sow, Doh Sanogo, Milagritos D. Tapia, Kathleen M. Neuzil, Karen L. Kotloff

| **Supplementary Table 1.** Adherence to WHO rehydration guidelines by dehydration severity among GEMS and VIDA cases in Mali | | | | | | |  |
| --- | --- | --- | --- | --- | --- | --- | --- |
|  | | **No dehydration** | | **Some dehydration** | | **Severe dehydration** | |
|  | | **GEMS**  **N=172** | **VIDA**  **N=32** | **GEMS**  **N=1616** | **VIDA**  **N=1454** | **GEMS**  **N=245** | **VIDA**  **N=117** |
| Adherent to WHO rehydration guidelines at home | Yes | 33 (19.2%) | 10 (31.2%) | -- | -- | -- | -- |
| Since the child developed diarrhea, how much have you been offering the child to drink? | More than usual | 72 (41.9%) | 22 (68.8%) | 1387 (85.8%) | 1158 (79.6%) | 183 (74.7%) | 99 (84.6%) |
| Since the child developed diarrhea, how much have you been offering the child to eat? | At least as much as usual | 102 (59.3%) | 18 (56.2%) | 430 (26.6%) | 569 (39.1%) | 81 (33.1%) | 50 (42.7%) |
| Adherent to WHO rehydration guidelines in a facility | Yes | 0 (0.0%) | 4 (12.5%) | 0 (0.0%) | 0 (0.0%) | 0 (0.0%) | 0 (0.0%) |
| Zinc prescribed | Yes | 0 (0.0%) | 4 (12.5%) | 4 (0.2%) | 80 (5.5%) | 0 (0.0%) | 9 (7.7%) |
| ORS administered | Yes | 1 (0.6%) | 1 (3.1%) | 12 (0.7%) | 6 (0.4%) | 8 (3.3%) | 4 (3.4%) |
| IV rehydration administered | Yes | 1 (0.6%) | 2 (6.2%) | 131 (8.1%) | 63 (4.3%) | 68 (27.8%) | 24 (20.5%) |
| ORS prescribed | Yes | 170 (98.8%) | 32 (100.0%) | 1606 (99.4%) | 1452 (99.9%) | 231 (94.3%) | 115 (98.3%) |
| Shaded cells indicate a treatment is required by WHO rehydration guidelines for the given degree of dehydration | | | | | | |  |

| **Supplementary Table 2.**  Adherence to WHO rehydration guidelines by dehydration severity among GEMS and VIDA cases in The Gambia | | | | | | | |
| --- | --- | --- | --- | --- | --- | --- | --- |
|  | | **No dehydration** | | **Some dehydration** | | **Severe dehydration** | |
|  | | **GEMS N=212** | **VIDA N=339** | **GEMS N=670** | **VIDA N=1154** | **GEMS N=143** | **VIDA N=148** |
| Adherent to WHO rehydration guidelines at home | Yes | 36 (17.0%) | 59 (17.4%) | -- | -- | -- | -- |
| Since the child developed diarrhea, how much have you been offering the child to drink? | More than usual | 90 (42.5%) | 103 (30.4%) | 243 (63.7%) | 899 (77.9%) | 69 (48.3%) | 108 (73.0%) |
| Since the child developed diarrhea, how much have you been offering the child to eat? | At least as much as usual | 113 (53.3%) | 231 (68.1%) | 407 (60.7%) | 571 (49.5%) | 70 (49.0%) | 70 (47.3%) |
| Adherent to WHO rehydration guidelines in a facility | Yes | 0 (0.0%) | 161 (47.5%) | 0 (0.0%) | 517 (44.8%) | 0 (0.0%) | 10 (6.8%) |
| Zinc prescribed | Yes | 0 (0.0%) | 161 (47.5%) | 0 (0.0%) | 581 (50.3%) | 0 (0.0%) | 39 (26.4%) |
| ORS administered | Yes | 10 (4.7%) | 61 (18.0%) | 92 (13.7%) | 861 (74.6%) | 19 (13.3%) | 84 (56.8%) |
| IV rehydration administered | Yes | 11 (8.8%) | 13 (6.1%) | 145 (26.0%) | 171 (25.5%) | 50 (41.0%) | 61 (42.7%) |
| ORS prescribed | Yes | 66 (31.1%) | 287 (84.7%) | 146 (68.9%) | 1137 (98.5%) | 159 (23.7%) | 135 (91.2%) |
| Shaded cells indicate a treatment is required by WHO rehydration guidelines for the given degree of dehydration | | | | | | |  |

| **Supplementary Table 3.** Adherence to WHO rehydration guidelines by dehydration severity among GEMS and VIDA cases in Kenya | | | | | | | |
| --- | --- | --- | --- | --- | --- | --- | --- |
|  | | **No dehydration** | | **Some dehydration** | | **Severe dehydration** | |
|  | | **GEMS**  **N=44** | **VIDA**  **N=83** | **GEMS**  **N=1023** | **VIDA**  **N=1050** | **GEMS**  **N=407** | **VIDA**  **N=414** |
| Adherent to WHO rehydration guidelines at home | Yes | 2 (4.5%) | 2 (2.4%) | -- | -- | -- | -- |
| Since the child developed diarrhea, how much have you been offering the child to drink? | More than usual | 13 (29.5%) | 5 (6.0%) | 357 (34.9%) | 361 (34.4%) | 48 (11.8%) | 66 (15.9%) |
| Since the child developed diarrhea, how much have you been offering the child to eat? | At least as much as usual | 13 (29.5%) | 48 (57.8%) | 219 (21.4%) | 336 (32.0%) | 47 (11.5%) | 115 (27.8%) |
| Adherent to WHO rehydration guidelines in a facility | Yes | 29 (65.9%) | 80 (96.4%) | 614 (60.0%) | 871 (83.0%) | 44 (10.8%) | 44 (10.6%) |
| Zinc prescribed | Yes | 29 (65.9%) | 80 (96.4%) | 618 (60.4%) | 910 (86.7%) | 187 (45.9%) | 365 (88.2%) |
| ORS administered | Yes | 40 (90.9%) | 64 (77.1%) | 999 (97.7%) | 710 (75.0%) | 387 (95.1%) | 64 (54.7%) |
| IV rehydration administered | Yes | 2 (4.5%) | 1 (0.9%) | 105 (10.3%) | 45 (4.8%) | 76 (18.7%) | 22 (18.8%) |
| ORS prescribed | Yes | 44 (100.0%) | 83 (100%) | 1006 (98.3%) | 1038 (98.9%) | 397 (97.5%) | 405 (97.8%) |
| Shaded cells indicate a treatment is required by WHO rehydration guidelines for the given degree of dhydration | | | | | | | |
